# Spatial Manipulation with Microfluidics

**DOI:** 10.3389/fbioe.2015.00039

**Published:** 2015-04-08

**Authors:** Benjamin Lin, Andre Levchenko

**Affiliations:** ^1^Department of Biomedical Engineering, Systems Biology Institute, Yale University, West Haven, CT, USA

**Keywords:** microfluidics, chemotaxis, gradients, cell migration, soft lithography

## Abstract

Biochemical gradients convey information through space, time, and concentration, and are ultimately capable of spatially resolving distinct cellular phenotypes, such as differentiation, proliferation, and migration. How these gradients develop, evolve, and function during development, homeostasis, and various disease states is a subject of intense interest across a variety of disciplines. Microfluidic technologies have become essential tools for investigating gradient sensing *in vitro* due to their ability to precisely manipulate fluids on demand in well-controlled environments at cellular length scales. This review will highlight their utility for studying gradient sensing along with relevant applications to biology.

## Introduction

**Figure 1:**
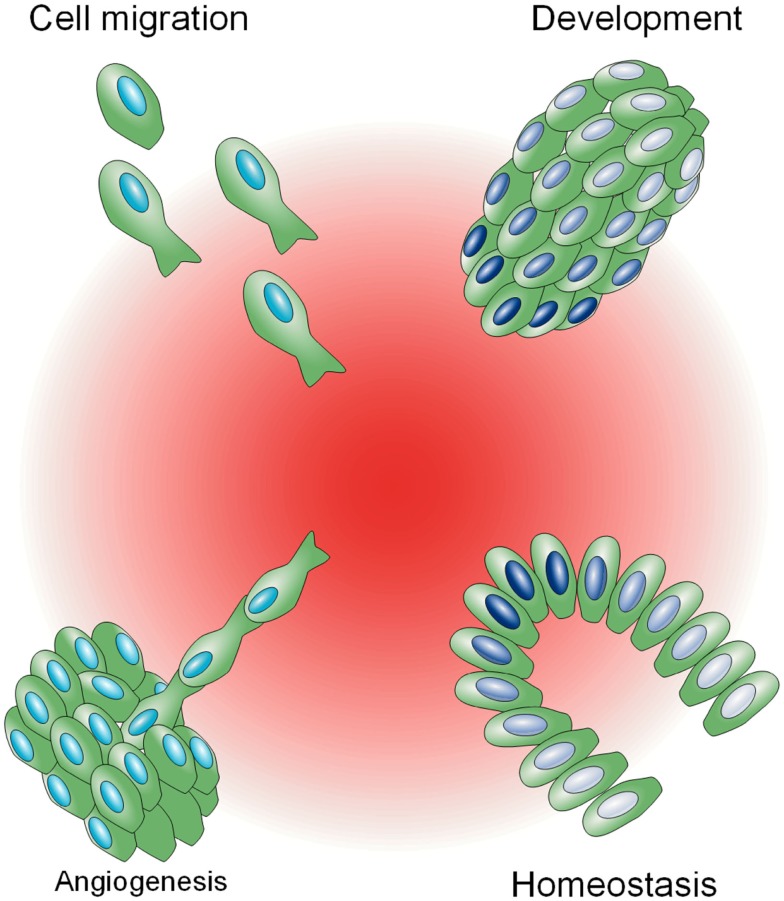
**Biological phenomena influenced by biochemical gradients**. A central spatial gradient of factors, shown in red, is depicted, influencing a variety of physiological processes. In clockwise order from the top left: cell migration toward a biochemical gradient (chemotaxis), different gene expression states, illustrated in gradations of blue, in relation to proximity to a gradient during development in a *Drosophila* embryo (top right) and in homeostasis in a colonic crypt (bottom right), and *de novo* blood vessel sprouting (angiogenesis).

Biochemical gradients are utilized in a variety of complex physiological processes as a mechanism to impart distinct signaling based on space (Figure 1). In the developmental biology field, diffusible morphogens, such as Bicoid (Driever and Nüsslein-Volhard, 1988) and Decapentaplegic (Dpp) (Ferguson and Anderson, 1992), exist as spatial gradients and are sufficient to induce spatial patterning in *Drosophila* embryos. In the cell migration field, a vast array of mammalian cells, including fibroblasts, leukocytes, epithelial, and endothelial cells, become motile in the presence of chemokines or growth factors, and display persistent, directed motion as single cells or collectives toward these molecules when they are spatially graded, a process known as chemotaxis (Singer and Kupfer, 1986). This innate ability to migrate directionally is utilized in immunity, wound healing, and angiogenesis, and is often exploited and selected for during metastatic progression (Roussos et al., 2011). In close analogy to migration, the growth of axon growth cones is biased toward or away from soluble and surface bound molecular gradients during neural patterning (Philipsborn and Bastmeyer, 2007). Last, homeostasis is maintained in the adult intestine by a spatial gradient of Wnt, which instructs transit-amplifying cells to proliferate and differentiate along the crypt axis (Gregorieff and Clevers, 2005).

Soluble biochemical gradients arise in biological systems largely through the diffusion of paracrine cell secretions and can disseminate distinct signals to adjacent cells based on their proximity to the gradient source. The mechanism in which biochemical gradients specify spatially diverse cellular decisions is often assumed to be through direct interpretation of perceived concentrations, which can induce phenotypes, such as differentiation and proliferation, upon crossing appropriate signaling thresholds, leading to sharp boundaries of cellular behavior. This is, however, an idealized situation under a steady state gradient. In complex *in vivo* environments, biochemical gradients may not reach a steady state, and consequently, cells may experience a time varying signal rather than a static dose and use temporal interpretation as a mechanism for decision making. More complexity arises when considering that cells may migrate and alter their spatial relationship with the gradient. Thus, when interpreting how biochemical gradients function, the spatial and temporal aspects of gradients must be carefully considered and controlled during experimentation.

Traditional approaches to studying biochemical gradients *in vivo* include familiar genetic knockdown and overexpression experiments to perturb native molecular gradients, as well as more actute perturbations, such as exogenously supplying molecules through microinjection to saturate an existing gradient or to introduce a gradient at a distal site. These latter approaches have close analogs *in vitro*, where, for example, appropriately positioned micropipettes have been used to eject molecules at a controlled frequency to produce an exponential gradient near cells of interest. However, several common issues arise during the application of these acute approaches: (1) the inputs themselves can vary between repetitions based on the equipment, i.e., the diameter of the micropipette, (2) once these perturbations are introduced, there is little subsequent dynamic control, and as a consequence, these spatial gradients vary over time, (3) the initial, environmental conditions of the biological substrates are poorly controlled, and (4) the introduced gradients are difficult to maintain for long durations. As alluded to above, these issues are undesirable from several vantage points: first, defining input–output relationships is inherently dependent on the fidelity of the inputs and second, both the spatial and temporal components of signaling gradients may each play a role in inducing subsequent phenotypes (Nahmad and Lander, 2011). The continued development of microfluidic devices has begun to address these issues. Microfluidic devices are now a widely popular tool to study gradients *in vitro*, due to their ability to manipulate fluids in a precise spatial-temporal manner at cellular length scales. Microfluidic devices have made several salient contributions to understanding gradient sensing, particularly in the field of cell migration, through their ability to accurately reproduce input gradients to create complex spatial profiles and to maintain graded inputs for extended periods of time. In this review, we begin with a brief introduction to soft lithography and continue by highlighting existing microfluidic technologies for gradient generation along with relevant applications to biological problems. We will conclude with future design considerations which are becoming increasingly relevant as these technologies become more widespread.

## Soft Lithography

Soft lithography is a set of techniques to fabricate micro and nanostructures based on replica molding with flexible elastomers (Xia and Whitesides, 1998) and is the basis for producing the majority of microfluidic devices. Templates for elastomers are produced by photolithography, a technique most widely known for creating integrated circuits, whereby photoresists are patterned onto silicon wafers using either laser printed transparent photomasks or chrome masks designed in computer assisted design (CAD) software. Sequential patterning of multiple photoresist layers can be used to produce structures with multiple heights onto the same template. Next, a polymer, such as polydimethylsiloxane (PDMS), is mixed with an appropriate crosslinker, poured onto the template and cured in an oven. After the curing process is complete, the PDMS device is removed from the template and now has the intended design features imprinted as hollow channels in its structure. The PDMS device is then bonded to a substrate, such as glass, thus forming sealed channels capable of housing fluids and cells. PDMS has several properties, which make it ideal for biological applications, including optical transparency down to ~300 nm, permeability to non-polar gases, and inertness (Whitesides et al., 2001), although recent work suggests that leaching of small uncured monomers into microchannel fluids and absorption of small, hydrophobic molecules from fluids may affect cell behavior (Regehr et al., 2009).

Microfluidic technologies are uniquely suited for precise fluid handling due to the laminar flow regime in which they operate. Laminar flow is predicted under a low Reynolds number, which is a dimensionless number defined by the ratio between inertial and viscous forces in a given system. As a consequence of laminar flow, when two fluids merge, they will remain separate and flow in parallel. Mass transport between the two fluids occurs purely by diffusion at the interface between the streams, thus enabling the predictable generation of concentration gradients, which has been exploited for investigating several biological phenomena. Below, we highlight various methodologies for producing gradients in microfluidics, beginning with a brief overview of general techniques followed by relevant biological applications. Microfluidic gradient generation devices can be generally divided into two categories: flow based devices where the biological samples of interest are exposed to flow and diffusion based devices where the biological samples of interest are housed in convection free environments. Their relative strengths and weaknesses will be discussed below.

## Flow Based Microfluidic Gradient Generators

**Figure 2:**
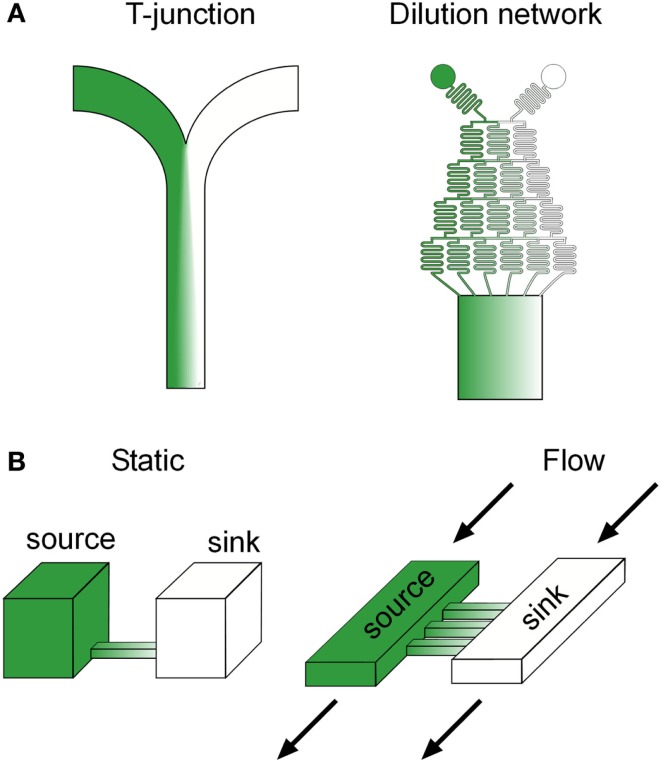
**Common microfluidic gradient generation designs**. **(A)** Flow based and diffusion based **(B)** microfluidic gradient generators. Green color represents the spatial distribution of a potential biochemical factor of interest in each device.

The most basic microfluidic design capable of gradient generation is a “T” or “Y” junction, which consists of two channels containing fluid inputs of different concentrations of a target molecule merging into a central channel (Figure 2A). Diffusion of the molecule occurs across the interface between the laminar streams as they merge into the central channel and the resulting spatial profile can be predicted based on the diffusion coefficient of the molecule and the location down the length of the central channel, which is correlated with the time the streams have been in contact (Brody and Yager, 1997). Although, in principle, “T” junctions can be used for investigating gradient sensing, the time to produce a smooth linear profile scales poorly with increases in the central channel width, owing to the slow diffusion of molecules from the central fluid interface to the channel edges. Thus for biological applications, “T” junctions have been primarily utilized to create sharp fluid boundaries, rather than smooth gradients, with fast flow rates that minimize diffusion, leading to applications such as sub-cellular (Takayama et al., 2001) and organism (Lucchetta et al., 2005) level binary patterning. These studies revealed new mechanistic insight into the lateral progression of epidermal growth factor (EGF) signaling (Sawano et al., 2002) as well as the signaling regulating developmental robustness in *Drosophila* (Lucchetta et al., 2005), providing good examples of research inherently dependent on microfluidic technology.

An alternative and now popular approach to microfluidic gradient generation, originally developed by the Whitesides group (Li Jeon et al., 2000), uses a branching network of serpentine channels reminiscent of a “Xmas” tree to serially dilute input streams into separate channels before merging the streams into a central channel (Figure 2A). This scheme dramatically decreases the time scale of gradient formation by interfacing multiple smaller laminar flow streams, as opposed to the two wide streams in “T” junctions, and can scale with central channel width by simply increasing the number of branches in the upstream flow network. Various complex gradient profiles can be dynamically produced in the downstream central channel through a combination of changing the number of inputs into the network, the relative flow rates of the inputs, and adding additional discrete branching networks (Dertinger et al., 2001). Flow splitting using parallel dividers has also been used to generate a diverse set of gradient profiles (Irimia et al., 2006).

Flow based microfluidic gradient generators (FBMGGs) were first utilized to explore natural biological phenomena in the seminal study done by Li Jeon et al. (2002), which investigated neutrophil chemotaxis to interleukin-8 (IL-8). As expected from prior studies, neutrophils directionally migrated up linear gradients of IL-8, with optimal chemotactic prowess close to the K_d_ of the IL-8 receptor. However, the ability to produce gradients of complex shape, such as a hill gradient, revealed that neutrophils could overshoot an IL-8 peak and migrate down a gradient, thus revealing new cellular behavior enabled by microfluidic devices. Since their first application to neutrophil chemotaxis, two essential properties of FBMGGs, the ability to maintain stable concentration gradients indefinitely and the ability to rapidly create gradients of complex, non-linear shape, have enabled several lines of new research. FBMGGs have been applied to study the directed migration/growth of a variety of cell types to different diffusible factors, such as breast cancer chemotaxis to EGF (Wang et al., 2004), endothelial cell chemotaxis to vascular endothelial growth factor A (VEGFA) (Barkefors et al., 2008), and growth cone turning toward brain-derived neurotrophic factor (BDNF) (Joanne et al., 2008). These studies were critically dependent on long-term stable gradients, as these cells migrate/grow appreciably on a several hour time scale. FBMGGs have also been applied to study other long term phenotypes, such as proliferation in fibroblasts (Park et al., 2009) and differentiation in human neural stem cells (Chung et al., 2005), by directly comparing cell phenotypes across a smooth gradient of applied factors. Last, FBMGGs have also been applied to study chemotaxis in single organisms, as demonstrated by Albrecht and Bargmann, who utilized a FBMGG to generate odorant gradients for studying chemotaxis in *Caenorhabditis Elegans* (Albrecht and Bargmann, 2011).

Beyond proof of concept experiments, several interesting biological insights have been revealed with FBMGGs. Tharp et al. (2006) discovered that human neutrophils could undergo chemorepulsion in IL-8 gradients with extremely high mean concentrations. Chemorepulsion was found to be dependent on protein kinase C (PKC), as gradients previously found to induce repulsion were converted to attraction with a PKC inhibitor. Herzmark et al. (2007) used a rapid, exponential gradient generating FBMGG to decouple temporal and spatial sensing during HL-60 (a neutrophil-like cell line) chemotaxis to f-Met-Leu-Phe (fMLP). They found that HL-60 cells could use a purely spatial mechanism to initiate chemotaxis and were more accurate with a greater fractional difference in concentration (change in concentration across cell length over mean concentration). Joanne et al. (2008) utilized a FBMGG to assess *Xenopus* spinal neuron growth cone turning to individual gradients of BDNF and laminin and in combination. Growth cones behaved as expected in individual gradients, with repulsion toward BDNF and attraction toward laminin, but showed complex behavior in combinatorial gradients. In conflicting gradients of laminin and BDNF, growth cones were attracted to laminin gradients and repelled away from the BDNF gradient at low mean BDNF concentrations, but showed the opposite response in conflicting gradients at a high mean BDNF concentration, indicating that the mean concentration of BDNF can dynamically tune growth cone responses.

One of the major drawbacks of FBMGGs is that flow occurs directly across cells anchored at the base of devices, and hence produces a shear stress on them. Flow induced shear introduces a mechanical input into experiments, which can influence cell behavior in chemotactic assays (Walker et al., 2005), and is sufficient by itself to bias cell migration (Polacheck et al., 2011). Although interstitial flow, or flow across tissues, can naturally occur across cells *in vivo* and thus may not be a completely foreign input (Swartz and Fleury, 2007), cells in these environments are anchored in a 3D extracellular matrix (ECM) and thus experience flow in a fundamentally distinct manner. To address this issue, a number of solutions have been developed to minimize shear stress, such as significantly increasing the height of flow chambers, housing cells in wells (Joanne et al., 2008), or separating the cell observation zone from the gradient generation chamber using a porous membrane (Chung et al., 2014). Another potential disadvantage of using FBMGGs is that cellular paracrine and autocrine signaling is continually washed away. Although this aids in more accurately defining cellular microenvironments based on the fluid inputs, these forms of cellular communication may be critical for the biological phenomena being investigated. For example, neutrophils secrete leukotriene B(4) as a relay molecule to enhance homing to formyl peptides across long distances (Afonso et al., 2012). As outlined below, pure diffusion based microfluidic gradient generators (DBMGGs) serve as an alternative technique to investigate gradient sensing where relevant cell secretions are retained and cells are not exposed to shear forces.

## Diffusion Based Microfluidic Gradient Generators

Diffusion based microfluidic gradient generators utilize passive diffusion between a source and a sink to generate spatial gradients across biological specimens in a convection free environment, and are particularly advantageous for cells with relatively low adhesiveness, cells in suspension, and cells normally residing in flow free environments (Figure 2B). As opposed to flow based gradient generators, spatial gradient profiles in diffusion based devices may take a substantial amount of time to reach steady state, depending on the distance and material between the source and sink, and thus, cells experience an initially evolving signal. Although alternative designs with minimal convection may alleviate this issue (Bhattacharjee et al., 2010), the majority of devices in use rely solely on passive diffusion, making these transients an important consideration when correlating subsequent phenotypes to the stimulus. Spatial profiles in diffusion based microfluidic generators can be manipulated by changing the connecting geometry between the source and the sink. For example, in rectangular channels, linear gradients will form at steady state, but can be sharper or shallower depending on the length of the channel (Paliwal et al., 2007). Non-linear gradient profiles can be produced by using geometries with unequal cross sectional interfaces with the source and sink, such as a trapezoid, which produce an unequal molecular flux (Mosadegh et al., 2007).

One of the potential problems when utilizing DBMGGs is cross flow between the source and sink, which may result, for example, from unequal fluid heights. Various strategies have been adapted to address this issue and are centered on increasing the fluidic resistance between the source and sink, such as decreasing the height of the connecting channel (Paliwal et al., 2007), integrating porous membranes which allow diffusion but resist flow (Abhyankar et al., 2006), and incorporating resistive 3D ECM gels (Mosadegh et al., 2007), which may better mimic *in vivo* conditions. Another potential problem, particularly in static DBMGGs, is a gradual change in gradient steepness due to saturation of the sink. This issue was addressed by Paliwal et al. (2007), who developed a continuous flow DBMGG, where the source and sink are continually replenished with dedicated fluid inputs (Figure 2B). Decreasing the height of the connecting channels and thus, increasing the fluidic resistance, was sufficient to prevent convective flux between the source and sink. In general, DBMGGs are slowly becoming more attractive to biologists, due to a growing trend toward simplistic designs which can be operated with a pipette and do not require sophisticated fluid automation. Several commercial solutions are now available, such as the IUVO chemotaxis assay plate from BellBrook labs and the μ-slide chemotaxis assay from Ibidi.

DBMGGs have been successfully utilized to investigate biochemical gradients at various cellular length scales, including single cells, various 3D cellular organizations, tissues, and organs. As with FBMGGs, single cell studies have been primarily geared toward studying chemotaxis, where a variety of spatial formats have been used to constrain migration to different spatial dimensions. In 1D designs, cells are housed in channels of constrained height and width, which induce uniaxial motility, whereas in 2D designs, cells migrate on a planar substrate in a large chamber, and last, in 3D designs, cell motility occurs within a 3D ECM scaffold. These spatial dimensions are important to consider when trying to correlate *in vitro* results to those *in vivo*, as recent work has indicated several fundamental differences between motility in 2D vs. 3D and indicate that 1D migration may be a good approximation (Doyle et al., 2009). Recent demonstrations of chemotaxis in DBMGGs include in 1D, Amobae chemotaxis to cAMP (Skoge et al., 2010), cancer cell chemotaxis to serum (Luo et al., 2014), neutrophil chemotaxis to fMLP (Irimia et al., 2007), mesenchymal stem cell chemotaxis to glioma conditioned medium (Smith et al., 2015), and HeLa chemotaxis toward synthetic molecules (Lin et al., 2012); in 2D, endothelial cell chemotaxis to vascular endothelial growth factor (Shamloo et al., 2008) and fibroblast chemotaxis to PDGF (Wu et al., 2012); and last in 3D, breast cancer cell chemotaxis to CXCL12 (Kim et al., 2013), dendritic cell chemotaxis to CCL19 (Haessler et al., 2009), and neurite turning toward or away from various factors (Kothapalli et al., 2011). Although many of these microfluidic studies have been carried out at the phenomenological level, recent studies have begun to yield new functional insight into the signaling pathways necessary to mediate chemotaxis and the physical mechanisms of cell migration. Notably, the Arp2/3 complex, an actin nucleator responsible for generating protrusions in migrating cells, was found to be dispensable for chemotaxis to PDGF in fibroblasts (Wu et al., 2012); chemotactic memory was demonstrated in social amebae using a gradient switching device (Skoge et al., 2014), a volume dependent mode of migration was uncovered under physical confinement (Stroka et al., 2014); and direct, spatially graded Rac activation was found to be sufficient to induce directed migration (Lin et al., 2012).

Multi-cellular, collective migration events, such as angiogenesis and collective cancer invasion, have also been investigated with passive diffusion devices. Nguyen et al. (2013) created a biomimetic, *in vitro* angiogenesis assay by first creating a pair of cylindrical lumens surrounded by a collagen gel by casting the gel in a PDMS casing around two enclosed needles. Removal of the needles created cylindrical hollow structures surrounded by ECM and human umbilical vein endothelial cells (HUVEC) were seeded into one structure to create a bioengineered vessel, while the other structure was used for supplying growth factors and other stimulants which emerged as gradients toward the vessel. Angiogenesis could be induced from the bioengineered vessel with a gradient of phorobol 12-myristate 13-acetate (PMA) and was significantly enhanced with a cocktail of appropriate factors. Other groups have also induced angiogenesis from a 2D endothelial cell interface into a 3D gel with a gradient of VEGF (Shin et al., 2011). Casavant et al. (2013) utilized microfluidic suspended capillary flow in an open system to create μdots of 3D ECM sandwiched by flow channels. Collective cancer cell invasion in 3D could be observed from prostate cancer cells seeded on top of the 3D ECM after a gradient of EGF was introduced from the bottom flow channel.

New complications arise when adapting microfluidic devices to studying biochemical gradients in tissues and organs, as they exist on a scale of hundreds of microns to millimeters, which is often incompatible with enclosed microfluidic gradient generators. Microfluidic designs utilizing open formats, extremely tall features, and reversible sealing have been adapted to study these larger substrates. Barkefors et al. (2009) designed an extremely tall (features on several hundred microns scale) passive gradient generation device, which could be sealed to a petri dish with a vacuum grid to study angiogenesis in whole embryonic kidneys and embryoid bodies. Directional angiogenesis could be readily observed in kidneys toward gradients of VEGFA, FGF2, and VEGFC after 48 h, and in embryoid bodies toward a gradient of VEGFA after 24 h. Günther et al. (2010) developed an open access device to directly interface with dissected mouse arteries under physiological pressure and temperature conditions. Arteries were loaded into an open well and subsequently transported and trapped into an observation chamber using fluid flow, where various drugs could be applied as gradients on either side of the vessel. A gradient of phenylephrine was sufficient to induce polarized vasoconstriction, suggesting a lack of lateral coupling between smooth muscle cells. Last, Queval et al. (2010) created a microfluidic probe capable of localized perfusion and aspiration of several input fluids to locally stimulate hippocampal brain slices in an open perfusion chamber accessible to fluorescent imaging. The utility of the device was shown by locally applying a fluorescent dye to a brain slice and tracking its distribution.

## Future Considerations

Microfluidic devices are increasingly being used to recapitulate complex biological phenomena influenced by biochemical gradients, due to their ability to accurately define cellular microenvironments in space and time over significant durations. In transitioning from perturbing cellular behavior on stiff 2D substrates to intricate 3D environments, microdevices have begun to achieve impressive levels of biomimicry. Complex multi-cellular architectures, such as vessels, tissues, and organs, are beginning to be incorporated into devices, enabling the exciting possibility of higher order systems analysis. Although the current trajectory of microfluidic devices is promising, several challenges need to be addressed to improve the relevancy of microfluidic device derived *in vitro* findings to *in vivo* settings. There is still substantial ambiguity surrounding both the time evolution and final spatial distributions of biochemical gradients *in vivo*. Acquisition of this data is imperative, because it informs what spatial-temporal inputs should be provided by microfluidic devices *in vitro*. The current, idealized spatial inputs used in many microfluidic devices (steady state gradients between a source and sink) could potentially be too artificial, and may elicit signaling and subsequent cellular behavior which may substantially deviate from that *in vivo*. The continued development of novel imaging tools will be needed to augment the currently limited pool of data surrounding these distributions. Another challenge is presented by the complex microenvironment in which cells reside. The vast majority of microfluidic studies have focused on a varying a single cue, predominantly a soluble chemokine or a combination of them; yet, cells in a physiological milieu constantly receive inputs from a variety of sources, such as the stiffness, chemical composition, and topography of the ECM, secretions from stromal cells, and oxygen tension. In principle, microfluidic devices are well suited for addressing how cells integrate combinatorial cues, as variables can be sequentially introduced and vetted in a defined setting. Last, potential readouts from microfluidic devices will need to be further exploited to transition from phenomenological observations to a mechanistic understanding of the underlying cellular signaling. A combination of fluorescent reporters, which can define intracellular protein activity and knowledge of the extracellular inputs will be crucial to making these findings.

## Conflict of Interest Statement

The authors declare that the research was conducted in the absence of any commercial or financial relationships that could be construed as a potential conflict of interest.
